# A Hyperspectral Image Classification Method Based on the Nonlocal Attention Mechanism of a Multiscale Convolutional Neural Network

**DOI:** 10.3390/s23063190

**Published:** 2023-03-16

**Authors:** Mingtian Li, Yu Lu, Shixian Cao, Xinyu Wang, Shanjuan Xie

**Affiliations:** 1 Institute of Remote Sensing and Earth Sciences, School of Information Science and Technology, Hangzhou Normal University, Hangzhou 311121, China; 2 SenseTime Research, Shenzhen 518000, China; 3 Zhejiang Provincial Key Laboratory of Urban Wetlands and Regional Change, Hangzhou Normal University, Hangzhou 311121, China

**Keywords:** hyperspectral image classification, multiscale convolutional neural network, nonlocal attention mechanism, feature fusion

## Abstract

Recently, convolution neural networks have been widely used in hyperspectral image classification and have achieved excellent performance. However, the fixed convolution kernel receptive field often leads to incomplete feature extraction, and the high redundancy of spectral information leads to difficulties in spectral feature extraction. To solve these problems, we propose a nonlocal attention mechanism of a 2D–3D hybrid CNN (2-3D-NL CNN), which includes an inception block and a nonlocal attention module. The inception block uses convolution kernels of different sizes to equip the network with multiscale receptive fields to extract the multiscale spatial features of ground objects. The nonlocal attention module enables the network to obtain a more comprehensive receptive field in the spatial and spectral dimensions while suppressing the information redundancy of the spectral dimension, making the extraction of spectral features easier. Experiments on two hyperspectral datasets, Pavia University and Salians, validate the effectiveness of the inception block and the nonlocal attention module. The results show that our model achieves an overall classification accuracy of 99.81% and 99.42% on the two datasets, respectively, which is higher than the accuracy of the existing model.

## 1. Introduction

The bandwidth of a hyperspectral image is usually tens of nanometers, much narrower than that of a multispectral image [1]. Therefore, the hyperspectral image has more abundant spectral information; it is widely used in various fields [2,3,4]. In the geoscience field, the correct pixel-level classification of remote-sensing images is the premise of many research tasks [5,6]. Hyperspectral remote-sensing images have natural advantages in classification tasks with rich spectral information [7]. Therefore, hyperspectral remote sensing is widely used in precision agriculture [8], rock and mineral identification [9], environmental monitoring [10], marine remote sensing [11], and other fields.

Traditional methods of classifying remote-sensing images include the classification of spectral features and the classification of statistical data features. The hyperspectral image classification method based on spectral features centers on the spectral curve of the optical property of the ground object to recognize the ground object. First, the spectral features of the hyperspectral image are extracted and transformed. Then, the pixels in the image are classified using the known spectral data in the spectral library and the matching algorithm. The representative method is spectral information angle mapping. The classification method based on the statistical characteristics of data uses the characteristics to establish a classification model by obtaining the statistics of each class on the training set and then classifying pixels with similar characteristics on the testing set to one class according to this statistic. The representative algorithms include the maximum likelihood and minimum distance methods.

With its rapid development, artificial intelligence has begun to play an important role in different engineering fields, such as in disease diagnosis [12]. The deep learning model has since emerged and become a widely studied research topic. In recent years, some deep learning models have been introduced into hyperspectral remote-sensing image classification. The stacked automatic encoder (SAE) [13] and deep belief network (DBN) [14] have been used to extract spectral information from hyperspectral images and achieve higher classification accuracy than traditional methods. Compared with SAE and DBN, a convolutional neural network (CNN) [15] is not limited by the input dimension. CNNs perform well in various tasks. For example, Yu et al. [16] proposed a vision-based automatic method for the surface condition identification of concrete structures, consisting of the most advanced pretrained CNN, transfer learning, and decision-level image fusion. It could accurately identify the crack contour through incorrect predictions of the limited area, proving its potential in practical applications. Similarly, LeBien et al. [17] proposed an end-to-end pipeline for training the CNN for the multi-species and multi-label classification of soundscape records, starting from the original and unmarked audio. The transfer learning of the pretraining model was used to reduce the necessary training data and time. The model achieved high classification accuracy in 24 species. In addition, CNNs have been widely used in image classification [18,19], object detection [20], semantic segmentation [21], and other fields.

CNNs are gradually being applied to the classification of hyperspectral images [22,23,24,25,26,27,28]. For example, Zhong et al. [29] designed a spatial–spectral residual network (SSRN) composed of spatial residual blocks and spectral residual blocks to jointly learn the spatial and spectral information in a hyperspectral image in order to further improve the recognition accuracy. Because the training time of an SSRN is too long, Wang et al. [30] designed a fast, dense spectral–spatial convolution (FDSSC) network that is faster than an SSRN and automatically extracts spatial features and spectral features in HSI by building dense spectral blocks and dense spatial blocks. Dense connections deepen the network and reduce the problem of gradient disappearance.

Multiscale features can better describe complex scenes; therefore, a multiscale strategy [31,32,33] is an effective way to improve the accuracy of HSI classification. Yang et al. [34] proposed a dual-channel convolutional neural network (two CNNs) to effectively extract images’ spectral and spatial features. The network uses different channels of a CNN to learn image features from the spectral and spatial dimensions. He et al. [35] proposed a multiscale 3D deep-convolution neural network (M3D-DCNN) for HSI classification, which can learn spatial features and spectral features together from hyperspectral image data in an end-to-end manner and then extract spectral information with a 1D-CNN. Pooja et al. [36] combined a multiscale strategy with the CNN network to achieve effective hyperspectral image classification, reduce the interference of adjacent pixels, and improve the performance of features. Wu et al. [37] proposed a multi-branch spectral–spatial joint network (MSSN) based on a CNN. The MSSN structure consists of two branches, each of which can extract the spectral and spatial features of hyperspectral images. Lee et al. [23] proposed a deep CNN with a deeper and broader context, which uses multiscale filter banks to obtain different receptive fields in order to extract the multiscale spectral–spatial fusion features of images. An inception block can make the network wider, reduce the number of parameters, and extract high-dimensional features. It uses convolution kernels of different receptive fields on the same layer of the network to extract features at different scales. Furthermore, an inception block is an effective means of solving the problem of incomplete multiscale feature extraction in hyperspectral image classification. Bei et al. [38] proposed a 3D asymmetric inception network (AINet) to overcome the over-fitting problem of hyperspectral image classification. AINet uses two asymmetric inception units, a spatial inception unit and a spectral inception unit, to effectively convey and classify features. In addition, they developed a data fusion transfer learning strategy to improve the model initialization and classification performance. Experiments showed that AINet was superior to all of the most advanced methods.

In addition to these factors, the attention mechanism has been widely used in computer vision in recent years. Lu et al. [39] proposed a new multiscale spatial–spectral residual network (CSMS-SSRN) based on 3D channels and spatial attention. The network uses different 3D convolution kernels to learn the frequency spectrum and spatial features from their respective residual blocks and then superimposes the extracted depth multiscale features into the 3D attention module to enhance the expressiveness of image features from both channels and spatial domains, thus improving the accuracy of classification. Because the SSRN and FDSSC networks require a large number of training samples to obtain good classification results, Sun et al. [40] proposed a special spatial attention network (SSAN), which combines simple spectral–spatial networks with attention mechanisms to extract the spectral and spatial characteristics of images. The nonlocal attention mechanism works well in video classification. It can also be applied to image classification, target detection, target segmentation, and other visual tasks, and the effect has been improved to varying degrees. A hyperspectral image is a kind of data similar to video, which is also applicable to nonlocal attention mechanisms. Hu et al. [41] combined a nonlocal attention mechanism with a CNN to present a multilevel progressive HSI SR network. The dense nonlocal and local block (DNLB) was constructed to combine local and global features, which are used to reconstruct super-resolution images at each level. They also developed a nonlocal channel attention block to extract the global features of HSIs efficiently. A number of experiments have demonstrated that their method could reconstruct hyperspectral images more accurately than existing methods.

Because the convolution kernels of the same layer are the same size, the problem of insufficient information extraction can easily occur. Focusing on the problems of spectral information redundancy and insufficient feature extraction at a single scale in the existing hyperspectral image classification research, this study proposes a CNN algorithm that combines a nonlocal attention module [42] and an inception block [43] to classify hyperspectral images. The nonlocal attention module can suppress the redundant information of hyperspectral images. Thus, the network can focus on essential features and use inception to extract and fuse multiscale spatial information to avoid insufficient spatial feature extraction on a single scale. This study conducted experiments on two hyperspectral datasets, Pavia University (PU) and Salinas (SA). The experiments showed that the proposed model could achieve higher classification accuracy than other deep learning models. The ablation experiments showed that adding inception and nonlocal attention mechanisms to the network effectively improved the model’s ability to extract spatial and spectral information from hyperspectral images.

The contributions of this study can be summarized as follows:

1. We use the inception and nonlocal attention mechanism to solve the problems of insufficient spatial–spectral feature extraction and the high redundancy of spectral information in hyperspectral images and to achieve higher classification accuracy;
2. We compare the nonlocal attention block with two other attention mechanisms to verify its effectiveness for hyperspectral image classification;
3. Experiments were conducted using other parameters that affect the classification accuracy of hyperspectral images with the deep learning model. The results provide a reference for further improving the classification accuracy of hyperspectral images.

Through undertaking this work, we hope to solve the problem of insufficient feature extraction for hyperspectral images and spectral feature extraction caused by spectral information redundancy. Moreover, we hope to further clarify the impact of different parameters on hyperspectral image classification, which will be helpful for follow-up research.

The remainder of this study is organized as follows. Section 2 introduces the principle of inception and the nonlocal attention mechanism. Section 3 introduces the structure of the proposed model. The comparison experiment and the discussion of the parameters that affect the method are presented in Section 4. Conclusions are given in Section 5.

## 2. Related Works

Inception and nonlocal blocks are commonly used in various computer vision tasks, and they also play an essential role in our network. These two structures are introduced below.

### 2.1. Inception Block

The increase in the depth and width of a network can improve its performance, but the cost of larger network parameters and a heavier load of calculation easily lead to overfitting. The inception structure proposed by Szegedy et al. [43] operates and integrates the feature map input from the previous layer by using convolution kernels and pooling operations of different sizes in the same layer to obtain a new feature map. This increases the width of the network but, at the same time, by the use of 1 × 1 convolution, it reduces the number of parameters and avoids excessive calculation in each layer. Therefore, the inception block is often used in computer vision studies. Many scholars have combined inception blocks with U-Net architecture and have proposed various image segmentation models. For example, Ibrahim et al. [44] added inception blocks to U-Nets to increase the network width and developed a new network structure aided by the feature extraction ability of inception blocks to improve building detection. Zhang et al. [45] integrated the inception block into U-Net, used the Res-inception module to replace the standard convolution layer to increase the width of the network, and used the inception block to extract features to build a deeper network structure and achieve higher performance than the existing algorithms. In addition, inception blocks have been applied in many computer vision applications, such as facial recognition [46], lithography hotspot detection [47], handwritten letter recognition [48], and breast cancer detection [49], with good results.

**Figure 1:**
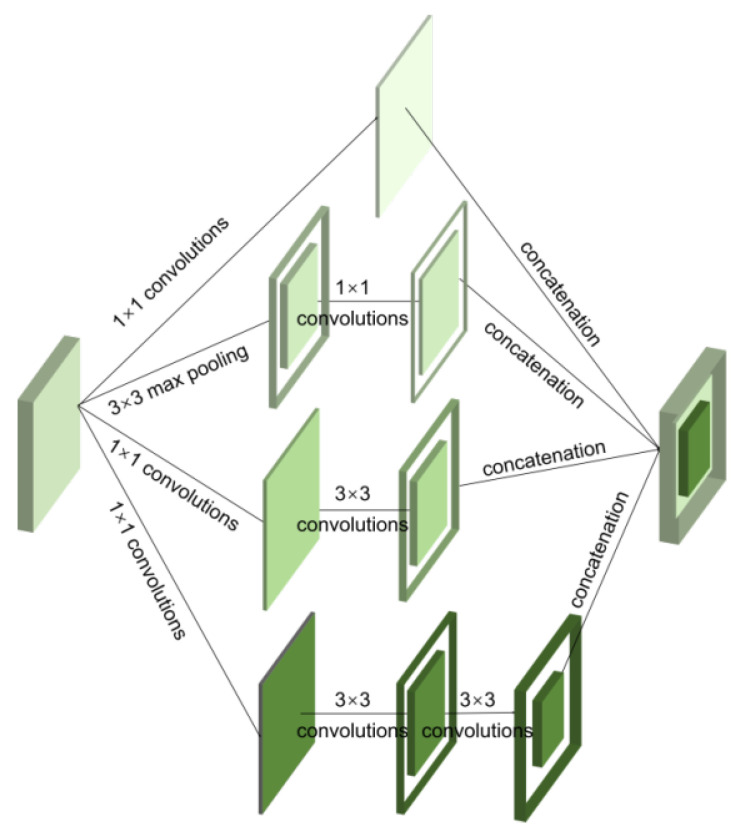
Structure of the inception V2 block.

Compared with the original inception, inception V2 uses two layers of 3 × 3 small convolution kernels instead of one layer of large 5 × 5 convolution kernels. This modification reduces the model parameters while keeping the receptive field unchanged, and it can provide more nonlinearity Figure 1 shows the structure of inception V2.

### 2.2. Nonlocal Block

**Figure 2:**
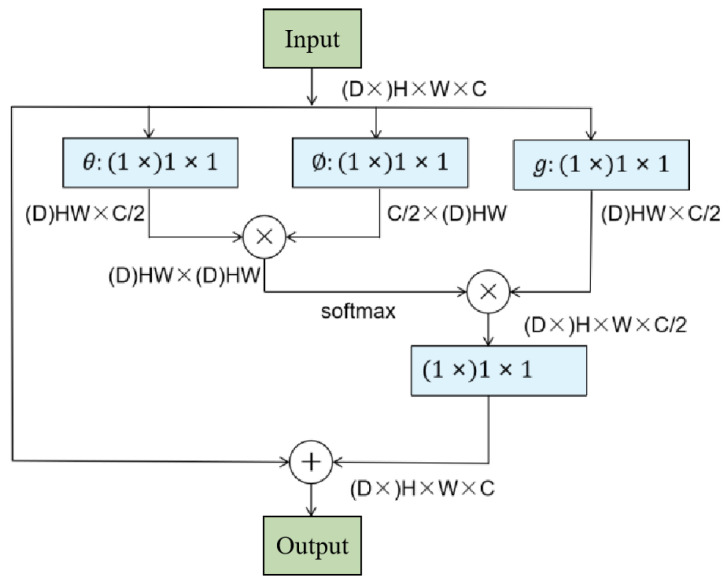
Nonlocal block structure. *g* is used to calculate the eigenvalue of the input data at the position, *θ* and *Ø* are two embeddings that are used to calculate similarity.

The attention mechanism is useful in image classification tasks and can make the model ignore irrelevant information and focus on key information. The nonlocal block was designed by Wang according to traditional nonlocal methods in computer vision. It can break the restriction that the convolution layer can only process adjacent elements. It makes the calculation of each pixel in the feature map connect with all other pixels in the whole feature map. It directly captures remote dependencies by calculating the interaction between any two positions on the image and obtaining global information. Figure 2 shows the structure diagram of the nonlocal block.

The nonlocal block performs very well in video classification, target detection, and other fields. Shokir [50] proposed a new nonlocal full-convolution network to capture global correlations more effectively for video saliency target detection obtained good results, proving the effectiveness of the nonlocal operation in saliency target detection. Quan [51] added the nonlocal block to a CNN for electrocardiogram classification. The electrocardiogram classification was significantly improved through nonlocal blocks to capture the long-term dependence of features in the spatial and channel domains. Wang [52] added an improved nonlocal block, called the asymmetric pyramid nonlocal block (APNB), to U-Net to automatically extract buildings from high-resolution aerial images. APNB captured global context information and improved the classification accuracy of pixels inside large buildings.

The formula for a nonlocal block is as follows:

(1) $$y_{i}=\frac{1}{c\left(X\right)}\sum_{\forall j}f\left(X_{i},X_{j}\right)g\left(X_{i}\right)$$

where *X* represents the input data (in this study, it refers to the three-dimensional image block of the input network), *y* represents the output data, and *i* and *j* represent the spatial positions of the input. *f* is a function used to calculate the similarity relationship with all other data, and g is used to calculate the eigenvalue of the input data at the position. c(X) is a normalization parameter. To simplify the problem, only the linear $\;g\left(X_{j}\right)$ case is considered; that is, $g\left(X_{j}\right)=W_{g}X_{j\;}.\;W_{g}\;$is a weight matrix that can be learned through training, which, depending on the input data, can be implemented in the neural network by the convolution operations of 1 × 1 or 1 × 1 × 1. There are many choices of functions. Here, we implement the embedded Gaussian, the formula for which is as follows:

(2) $$f\left(X_{i},X_{j}\right)=e^{\theta {\left(X_{i}\right)}^{T}\emptyset \left(X_{j}\right)}$$

where $\theta \left(X_{i}\right)=W_{\theta }X_{i}$, and $\emptyset \left(X_{j}\right)=W_{\theta }X_{j}\;$is also achieved by convolution operations of 1 × 1 or 1 × 1 × 1 on normalized parameters$\;C\left(X\right)=\sum_{\forall }f\left(X_{i},X_{j}\right)$. As such, y can be fully expressed as follows:

(3) $$y=softmax\left(X^{T}W_{\theta }^{T}W_{\emptyset }X\right)g\left(X\right)$$

## 3. The Proposed Method

To solve the problems of insufficient spatial feature extraction at a single scale, the presence of many bands, and the high redundancy of spectral information in hyperspectral image classification, we introduce the inception and nonlocal attention mechanisms into the CNN. Unlike the existing models, our proposed model uses several convolution kernels of different sizes on the same layer of the network to extract features, and the inception block provides multiscale receptive fields for the network to make feature extraction more efficient. The nonlocal attention mechanism is not limited to adjacent pixels and can determine the correlation between any positions, which is equivalent to constructing a convolution kernel of the size of a feature map. Therefore, the network can extract more comprehensive spatial and spectral features while suppressing the redundant information between spectral bands.

**Figure 3:**
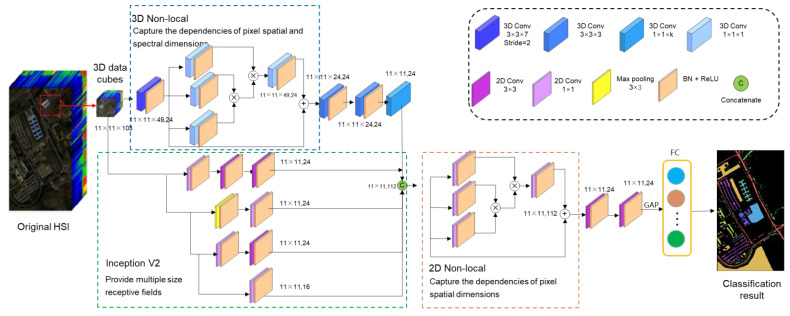
Proposed network structure.

The network structure of this study is shown in Figure 3 First, the hyperspectral image is processed into several small overlapping H × W × C data cubes, which are the inputs of the network. The front end of the model can be considered as two branches. One is a 3D CNN with a nonlocal attention module responsible for extracting spatial–spectral information from input data. The nonlocal modules are similar to the receptive field of the extended convolution kernel. Nonlocal operations capture the remote correlation directly by calculating the interaction between any two locations, rather than being limited to adjacent points. This is equivalent to constructing a convolution kernel as large as the feature map to obtain more information. At the same time, the nonlocal block can also capture the long-distance interaction between pixels in different bands, which can better use the rich spectral information of hyperspectral images. The nonlocal attention module can also help the model to suppress irrelevant information and pay more attention to the salient features, thereby enhancing the ability of the network to extract spatial and spectral features.

Hyperspectral images are 3D data with spatial and spectral dimensions according to different input data dimensions. The nonlocal operations can work in the spatial dimension, which is called the 2D nonlocal attention module when the input data dimension is H × W × C. When the input data dimension is D × H × W × C, a nonlocal operation can play a role in the spectral dimension, and it is called the 3D nonlocal attention module.

Because the input data have not been processed by dimension reduction or band selection, the spectral dimension contains much redundant information. Therefore, the input data first pass through a layer of spectral dimensions with a step size of 2 and a convolution kernel size of 3 × 3 × 7 to gather spectral information and simultaneously extract spatial–spectral joint features of input data to enhance salient features and suppress redundant data through 3D nonlocal modules. Then, we use a 2-layer 3 × 3 convolution layer to further extract spatial features and a 1-layer 1 × 1 × K convolution to obtain a 2D feature map.

Another branch is the multiscale spatial feature fusion module based on the inception structure. This module uses convolution kernels of different sizes to extract features of different scales. All these features are fused through the concat operation to finally obtain multiscale spatial features. Through the multiscale spatial feature fusion module, spatial features of different sizes are extracted and fused, and the features of the input data on each scale become more prominent. The data of the two branches are fused, and then the salient features are further enhanced through a 2D nonlocal attention module. After that, a 3 × 3 convolution and a global averaging pooling layer are applied on the feature map, and a SoftMax classifier is used for the final classification.

## 4. Ablation Study

### 4.1. Experimental Data and Evaluation Metrics

**Figure 4:**
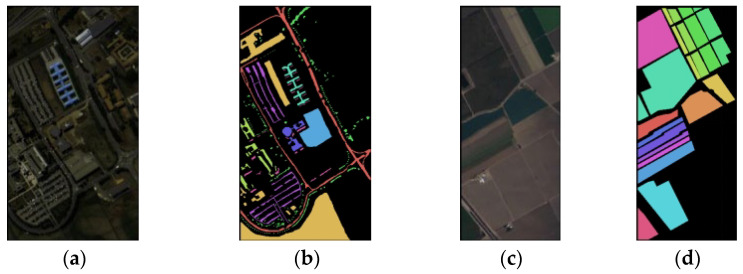
PU and SA dataset pseudo-color images and ground truth: (**a**) pseudo-color image of PU, (**b**) ground truth of PU, (**c**) pseudo-color image of SA, (**d**) ground truth of SA.

To verify the effectiveness of the network and the influence of other variables on the classification results of hyperspectral images, we used two published hyperspectral image datasets, Salinas (SA) and Pavia University (PU) [53], as experimental data. The datasets are shown in Figure 4.

This study used three quantitative indicators to assess the merits of the classification results: overall accuracy (OA), average accuracy (AA), and the Kappa coefficient. Among them, the OA refers to the proportion of the number of correctly classified samples on the test set to the total samples of the test set; the AA is the sum of the number of samples in each category to the proportion of the population sample, which is then divided by the total number of categories to obtain the AA. The Kappa coefficient measures the model classification accuracy for the consistency of the model prediction classification and the actual classification. The value is generally between 0 and 1; the larger the value, the higher the classification accuracy.

### 4.2. Experimental Environment and Parameter Settings

All experiments were conducted on the same Dell laptop made in Xiamen, China, configured with an Intel(R) Core ™ i5-6300HQ CPU @ 2.3 GHz, 16.0 GB of running memory (Santa Clara, CA, USA), and an NVIDIA GeForce GTX 960 graphics card (Santa Clara, CA, USA). The operating system was Windows 10, and the deep learning framework was Pytorch 1.6.0 and CUDA 10.1.

The initialization of the convolutional kernel and fully connected layer parameters in the network adopted the He normal method, the initial bias was 0, and the network was trained with the Adam optimizer. The learning rate was set to 0.001, the batch size was 32, and the number of epochs was 100.

The training and test image samples were selected by random sampling. On the whole hyperspectral image, the corresponding proportion of pixels was randomly selected as the training set for model training, and the remaining pixels were selected as the test set. In the training set, 10% of the samples were selected as the validation set for parameter optimization. To ensure that the pixels at the edge of the image can be selected, we set the fill appropriately for the size of the input data around the image.

### 4.3. Experiment

#### 4.3.1. Selection of the Network Backbone

The network backbone could have been a 2D CNN, 3D CNN, or 3D–2D CNN. Compared with the 2D CNN, a 3D CNN has advantages in hyperspectral datasets but is more computation intensive. Using a 3D–2D CNN considers both the classification effect and the amount of calculation. Table 1 shows the classification performance of the three network structures on two hyperspectral datasets.

Table 1 shows that, on the PU and SA datasets, the OA of a 3D CNN with the same number of layers is higher than that of a 2D CNN. However, the number of parameters of the network is also greater, and the training time is several times that of a 2D CNN. The number of parameters of a 3D–2D CNN is significantly lower than a 3D CNN. Even with many bands, the number of parameters is lower than for a 2D CNN, reducing the risk of model overfitting. Moreover, the training time of a 3D–2D CNN is also significantly shorter. Additionally, the overall classification accuracy of the 3D–2D model is comparable to that of the 3D model on the PU dataset and is even better than that of the 3D CNN on the SA dataset. Thus, we selected the 3D–2D CNN as the backbone.

#### 4.3.2. Comparing the Effectiveness of Multiscale Attention Modules

To verify the effectiveness of the multiscale spatial feature fusion module (MS) and nonlocal attention module added in this study, we added the multiscale spatial feature fusion module and 2D and 3D nonlocal attention modules to the network in turn. Table 2 shows the classification accuracy of several model structures on the PU and SA datasets.

It shows that, on the PU dataset, when an MS and a nonlocal module are added to the baseline model, the overall classification accuracy of the model is improved by 0.163% and 0.294%, respectively, indicating that the two modules improve the performance of the model. The nonlocal and MS modules are added to the network at the same time. Table 2 presents three different forms: adding 2D nonlocal and 3D nonlocal separately and adding 2D and 3D nonlocal simultaneously. Regarding classification accuracy, adding nonlocal and MS to the network simultaneously produces better results than adding them separately. The performance of the 3D nonlocal module is better than that of the 2D nonlocal module, and the simultaneous use of the 2D and 3D nonlocal modules further improves the model’s performance, indicating that the simultaneous use of the attention mechanism in the spatial and spectral dimensions achieves better results.

In addition to the nonlocal attention mechanism, SENet [54] and CBAM [55] are also high-quality attention mechanisms that are used in image classification. Therefore, we compared the effects of three attention mechanisms on the classification of two hyperspectral images. Table 2 also shows the OA of the network structure with three attention modules on the PU and SA datasets.

From the results shown in Table 2, we can see that the classification performance of the three network structures with attention modules on the two datasets is slightly different from that of the baseline. For the PU dataset, the classification accuracy is improved with three different attention modules, where the accuracy levels of SE and CBAM are similar, and the nonlocal module gives the best performance. For the SA dataset, because the number of bands is further increased, and the similarity of various ground features in the SA dataset is higher than that in the PU dataset, the classification accuracy with SE and CBAM is decreased, while the classification accuracy of the model with a nonlocal module is still steadily improved. Therefore, we selected the nonlocal module, which can capture the long-distance correlation between spectra and can better improve classification accuracy, as the final attention block.

#### 4.3.3. Searching for the Optimal Parameters

In this section, the parameters affecting the classification accuracy of hyperspectral images are discussed, including the number of convolution kernels, the size of neighboring pixel blocks, and the rations of training samples.

1. The number of convolutional kernels

The number of convolutional kernels in the network structure is an important parameter for determining the appropriate number of convolutional kernels. Each layer in the model uses the same number of convolutional kernels. Table 3 shows the OA and the number of parameters of the model on the SA and PU datasets when using different numbers of convolutions.

When there are 12 convolutional kernels, the model’s classification accuracy on the PU and SA datasets is 98.962% and 96.640%, respectively. The classification accuracy of the model on the two datasets is significantly improved by gradually increasing the number of convolutional kernels. When the number of convolutional kernels increased from 24 to 30, the OA of the model on the PU and SA datasets increased by only 0.084% and 0.054%, respectively, the feature extraction ability of the model became saturated, while the parameters increased by 31.67% and 30.72%, respectively. It is uneconomical to increase the number of convolutional kernels in the model, so the final determination of the number of convolutional kernels in the model is 24.

2. Neighboring pixel block size and proportion of training samples

Since the input of the model is a neighborhood pixel block extracted from a hyperspectral image, the size of the neighborhood pixel block determines the amount of data received by the model, which has a significant impact on the final classification accuracy. Moreover, the proportion of training samples used also affects the effect of model feature extraction; therefore, we used 5%, 10%, 15%, and 20% of the training sample ratios to set 5 spatial sizes (5, 7, 9, 11, and 13) on the experimental dataset to explore the influence of the neighborhood pixel block size and training sample ratio on the classification accuracy. The results are shown in Table 4.

Table 4 shows that, as the size of the neighborhood pixel block increases from 5 to 13, the classification performance of the model on both datasets also improves, and this pattern persists when the proportion of training data changes. This is because larger neighborhood blocks provide more information and allow the model to extract more distinguishing features. However, it should also be noted that the classification accuracy of the model is highest when the size is 11, and further increasing the size of the neighborhood pixel block reduces the classification accuracy. For the proportion of training samples, the classification accuracy can be significantly improved by increasing the number of training samples but, like the neighborhood pixel block size, there is also a maximum value of 15%; the classification accuracy of this value is basically stable, and the classification accuracy of the training data will decrease when the proportion of training samples is further increased.

#### 4.3.4. Algorithm Comparison Experiments

To verify the effectiveness of the proposed model (2-3D-NL CNN), we compared the model with the 2D CNN, 3D CNN, HybridSN [25],Two-CNN [32], SSRN [29], SSAN [40], FDSSC [30], Hamida [56], PResNet [57], and M3D-DCNN [35] models. Each model was trained with 10% of the training samples, and the spatial size of the input data was 5. Table 5 presents the parameter settings of each model. Table 6 presents the classification performance of several algorithms on the PU and SA datasets. It can be seen from Table 6 that the 2-3D-NL CNN performs better than other models in OA and AA, which proves the advanced state of the model. The 2-3D-NL CNN achieved higher classification accuracy than FDSSC, SSRN, PResNet, and other models on the PU and SA datasets because the 2-3D-NL CNN made full use of the multiscale spatial features, used a nonlocal attention mechanism to effectively capture the correlation between spectra, and finally extracted more discriminative spatial and spectral features.

**Figure 5:**
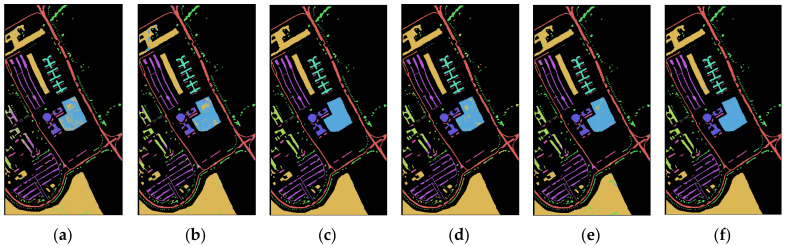

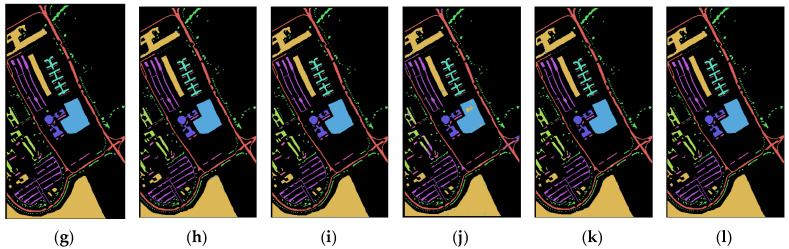
Classification effect of different algorithms on the PU dataset: (**a**) 2D-CNN, (**b**) 3D-CNN, (**c**) HybridSN, (**d**) Two-CNN, (**e**) Hamida, (**f**) PResNet, (**g**) M3D-DCNN, (**h**) FDSSC, (**i**) SSRN, (**j**) SSAN, (**k**) 2-3D-NL CNN. (**l**) The ground truth.

**Figure 6:**
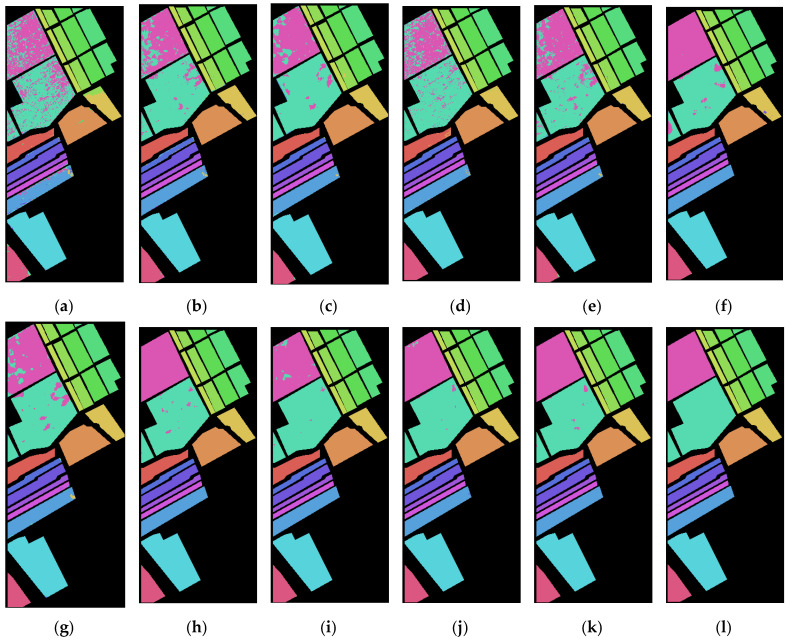
Classification effect of different algorithms on the SA dataset: (**a**) 2D-CNN, (**b**) 3D-CNN, (**c**) HybridSN, (**d**) Two-CNN, (**e**) Hamida, (**f**) PResNet, (**g**) M3D-DCNN, (**h**) FDSSC, (**i**) SSRN, (**j**) SSAN, (**k**) 2-3D-NL CNN. (**l**) The ground truth.

Figure 5 and Figure 6 are the classification effect diagrams of the algorithms. From the classification map of the two datasets, it is clear that simple 2D CNNs and 3D CNNs have obvious pixel misclassification owing to their insufficient feature extraction capabilities. The performance of several other models is much better, and the 2-3D-NL CNN proposed in this paper achieved the best classification effect; almost no pixels were misclassified on the PU dataset. The classification map obtained for the SA dataset is smoother that of other models, and there are fewer pixels misclassified.

## 5. Conclusions

Hyperspectral image classification is an important research area in the field of hyperspectral remote sensing. The hyperspectral image classification method based on CNN performs well in the hyperspectral image classification task. However, a fixed size of convolution kernel often leads to inadequate feature extraction, and the information redundancy of spectral dimension also makes spectral feature extraction difficult. To solve the above-mentioned problems, we proposed a 2-3D NL CNN. Based on the convolution neural network, an inception block and a nonlocal attention mechanism are introduced to improve the classification accuracy of hyperspectral images. Experiments were carried out based on the PU dataset and the SA dataset. The results indicate the following:

1. The 2-3D NL CNN effectively improves the classification accuracy of hyperspectral images. The inception block uses convolution kernels of different sizes to provide different sizes of receptive fields for the network, making feature extraction more comprehensive. The nonlocal attention mechanism enhances the spectral feature extraction ability of the network and suppresses the information redundancy of spectral dimension.
2. The nonlocal attention mechanism is more suitable for hyperspectral image classification tasks. Our experiment compared three attention mechanisms, namely SENet, CBAM, and a nonlocal attention mechanism, and the nonlocal attention mechanism improved the classification accuracy more significantly for the two datasets. This is mainly because the nonlocal attention mechanism can factor in the correlation between the pixels at a greater distance, as well as accounting for the pixels to be classified.

Although the model proposed in this paper showed excellent performance in hyperspectral image classification, it still has some shortcomings. For example, the spectral features of the pixels to be classified are actually disturbed by the spectral information of the neighboring pixels, and the generalization ability of the model was not verified. Future work should pay more attention to how to extract spatial features while avoiding the interference caused by the spectral information of neighboring pixels to the spectral feature extraction of classified pixels. Furthermore, more attention should be paid to the generalization ability of the model to find new hyperspectral image datasets for experiments.

## Figures and Tables

**Table 1:** Comparison of three network structures.

Model	OA (%)		Training Time (s)		Number of Parameters	
	PU	SA	PU	SA	PU	SA
**2D**	98.663	97.038	152.141	210.043	48,850	70,841
**3D**	99.149	97.270	815.031	1784.013	90,322	90,497
**3D–2D**	99.132	98.156	556.057	1093.856	57,490	57,665

**Table 2:** Classification results of different methods on the Pavia University (PU) and Salinas (SA) datasets.

Method	MS	2D Nonlocal	3D Nonlocal	SE	CBAM	OA (%)	
						PU	SA
**baseline**						99.132	98.156
**Ms**	√					99.295	98.254
**Nonlocal**		√	√			99.426	98.294
**Ms+2D nonlocal**	√	√				99.439	98.449
**Ms+3D nonlocal**	√		√			99.518	98.525
**Ms+nonlocal**	√	√	√			**99.592**	**98.567**
**MS+SE**	√			√		99.349	97.922
**MS+CBAM**	√				√	99.332	97.893

**Table 3:** OA and parameters with different convolution kernels.

Dataset	Performance	Number of Convolutional Kernels			
		12	18	24	30
**PU**	**OA (%)**	98.962	99.275	99.533	99.617
	**Parameters**	67,694	93,158	125,832	165,686
**SA**	**OA (%)**	96.640	97.764	98.290	98.344
	**Parameters**	71,825	97,331	130,037	169,943

**Table 4:** OA (%) for different neighboring pixel block sizes and different proportions of training datasets.

Datasets	Proportion	Neighboring Pixel Block Size				
		5	7	9	11	13
**PU**	**5**	98.736	99.553	99.718	**99.731**	98.729
	**10**	99.579	99.893	99.924	**99.927**	99.915
	**15**	**99.731**	**99.935**	**99.948**	**99.989**	**99.972**
	**20**	99.718	99.922	99.934	**99.961**	99.952
**SA**	**5**	93.892	98.264	98.578	**98.582**	98.554
	**10**	97.257	99.218	99.589	**99.809**	98.756
	**15**	**98.598**	**99.465**	**99.624**	**99.682**	**99.635**
	**20**	98.454	99.463	99.587	**99.645**	99.627

**Table 5:** Parameter settings of each model.

Method	Conv Nb	Spatial Size	FC Nb	Parameter Nb
2D CNN	6	5	1	1152
3D CNN	6	5	1	4176
HybridSN	4	5	2	3520
Two-CNN	5	5	2	6454
Hamida	4	5	2	40,740
PResNet	10	5	1	2468
M3D-DCNN	4	5	1	2528
FDSSC	9	5	1	46,308
SSRN	11	5	1	45,688
SSAN	5	5	1	26,880
2-3D-NL CNN	6	5	1	4786

**Table 6:** Classification results of different methods on the PU and SA datasets.

Method	PU Dataset		SA Dataset	
	OA(%)	AA(%)	OA(%)	AA(%)
2D CNN	90.85 ± 0.37	87.68 ± 0.32	91.01 ± 0.28	92.37 ± 0.25
3D CNN	93.80 ± 0.24	89.85 ± 0.27	93.86 ± 0.25	92.75 ± 0.18
HybridSN	97.33 ± 0.19	97.16 ± 0.15	97.44 ± 0.22	97.32 ± 0.19
Two-CNN	94.63 ± 0.27	93.31 ± 0.22	91.38 ± 0.36	89.74 ± 0.43
Hamida	94.51 ± 0.42	93.68 ± 0.35	93.15 ± 0.34	92.86 ± 0.28
PResNet	99.76 ± 0.21	99.68 ± 0.23	99.59 ± 0.25	99.45 ± 0.28
M3D-DCNN	98.98 ± 0.34	98.34 ± 0.28	98.78 ± 0.35	98.55 ± 0.32
FDSSC	99.56 ± 0.18	99.35 ± 0.25	99.45 ± 0.28	99.34 ± 0.34
SSRN	99.42 ± 0.26	99.35 ± 0.32	99.22 ± 0.26	99.31 ± 0.33
SSAN	99.64 ± 0.34	99.54 ± 0.28	99.35 ± 0.25	99.27 ± 0.29
**2-3D-NL CNN**	**99.81** ± **0.25**	**99.76** ± **0.24**	**99.65** ± **0.28**	**99.42** ± **0.25**

## Data Availability

Dataset details and downloads are available at https://www.ehu.eus/ccwintco/index.php?title=Hyperspectral_Remote_Sensing_Scenes (accessed on 13 March 2023).

## References

[1] Huang R., He M. (2005). Band Selection Based on Feature Weighting for Classification of Hyperspectral Data. IEEE Geosci. Remote Sens. Lett..

[2] Li J., Khodadadzadeh M., Plaza A., Jia X., Bioucas-Dias J.M. (2016). A Discontinuity Preserving Relaxation Scheme for Spectral–Spatial Hyperspectral Image Classification. IEEE J. Sel. Top. Appl. Earth Obs. Remote Sens..

[3] Liang L., Di L., Zhang L., Deng M., Qin Z., Zhao S., Lin H. (2015). Estimation of crop LAI using hyperspectral vegetation indices and a hybrid inversion method. Remote Sens. Environ..

[4] Yang X., Yu Y. (2017). Estimating Soil Salinity Under Various Moisture Conditions: An Experimental Study. IEEE Trans. Geosci. Remote Sens..

[5] Feddema J.J., Oleson K.W., Bonan G.B., Mearns L.O., Buja L.E., Meehl G.A., Washington W.M. (2005). The Importance of Land-Cover Change in Simulating Future Climates. Science.

[6] Li S.H., Liu X., Li X.P., Chen Y.M. (2017). Simulation model of land use dynamics and application: Progress and prospects. J. Remote Sens..

[7] Ding X., Zhang S., Li H., Wu P., Dale P., Liu L., Cheng S. (2020). A restrictive polymorphic ant colony algorithm for the optimal band selection of hyperspectral remote sensing images. Int. J. Remote Sens..

[8] Lu B., Dao P., Liu J., He Y., Shang J. (2020). Recent Advances of Hyperspectral Imaging Technology and Applications in Agriculture. Remote Sens..

[9] van Ruitenbeek F., van der Werff H., Bakker W., van der Meer F., Hein K. (2019). Measuring rock microstructure in hyperspectral mineral maps. Remote Sens. Environ..

[10] Pipitone C., Maltese A., Dardanelli G., Brutto M.L., La Loggia G. (2018). Monitoring Water Surface and Level of a Reservoir Using Different Remote Sensing Approaches and Comparison with Dam Displacements Evaluated via GNSS. Remote Sens..

[11] Grotte M.E., Birkeland R., Honore-Livermore E., Bakken S., Garrett J.L., Prentice E.F., Sigernes F., Orlandic M., Gravdahl J.T., Johansen T.A. (2021). Ocean Color Hyperspectral Remote Sensing With High Resolution and Low Latency—The HYPSO-1 CubeSat Mission. IEEE Trans. Geosci. Remote Sens..

[12] Sallam N.M., Saleh A.I., Ali H.A., Abdelsalam M.M. (2023). An efficient EGWO algorithm as feature selection for B-ALL diagnoses and its subtypes classification using peripheral blood smear images. Alex. Eng. J..

[13] Chen Y., Lin Z., Zhao X., Wang G., Gu Y. (2014). Deep Learning-Based Classification of Hyperspectral Data. IEEE J. Sel. Top. Appl. Earth Obs. Remote Sens..

[14] Chen Y., Zhao X., Jia X. (2015). Spectral–Spatial Classification of Hyperspectral Data Based on Deep Belief Network. IEEE J. Sel. Top. Appl. Earth Obs. Remote Sens..

[15] Krizhevsky A., Sutskever I., Hinton G.E. (2017). Imagenet classification with deep convolutional neural networks. Commun. ACM.

[16] Yu Y., Samali B., Rashidi M., Mohammadi M., Nguyen T.N., Zhang G. (2022). Vision-based concrete crack detection using a hybrid framework considering noise effect. J. Build. Eng..

[17] LeBien J., Zhong M., Campos-Cerqueira M., Velev J.P., Dodhia R., Ferres J.L., Aide T.M. (2020). A pipeline for identification of bird and frog species in tropical soundscape recordings using a convolutional neural network. Ecol. Inform..

[18] He K., Zhang X., Ren S., Sun J. Deep residual learning for image recognition. Proceedings of the IEEE Computer Society Conference on Computer Vision and Pattern Recognition (CVPR).

[19] Yu L., Chen H., Dou Q., Qin J., Heng P.A. (2017). Automated Melanoma Recognition in Dermoscopy Images via Very Deep Residual Networks. IEEE Trans. Med. Imaging.

[20] Ren S., He K., Girshick R., Sun J. Faster R-CNN: Towards real-time object detection with region proposal networks. Proceedings of the 29th Annual Conference on Neural Information Processing Systems (NIPS).

[21] Chen L.-C., Papandreou G., Kokkinos I., Murphy K., Yuille A.L. (2018). DeepLab: Semantic Image Segmentation with Deep Convolutional Nets, Atrous Convolution, and Fully Connected CRFs. IEEE Trans. Pattern Anal. Mach. Intell..

[22] Chen Y., Jiang H., Li C., Jia X., Ghamisi P. (2016). Deep Feature Extraction and Classification of Hyperspectral Images Based on Convolutional Neural Networks. IEEE Trans. Geosci. Remote Sens..

[23] Lee H., Kwon H. (2017). Going Deeper With Contextual CNN for Hyperspectral Image Classification. IEEE Trans. Image Process..

[24] Li Y., Zhang H., Shen Q. (2017). Spectral–Spatial Classification of Hyperspectral Imagery with 3D Convolutional Neural Network. Remote Sens..

[25] Roy S.K., Krishna G., Dubey S.R., Chaudhuri B.B. (2020). HybridSN: Exploring 3-D–2-D CNN Feature Hierarchy for Hyperspectral Image Classification. IEEE Geosci. Remote Sens. Lett..

[26] Yu S., Jia S., Xu C. (2017). Convolutional neural networks for hyperspectral image classification. Neurocomputing.

[27] Yuan Q., Zhang Q., Li J., Shen H., Zhang L. (2019). Hyperspectral Image Denoising Employing a Spatial–Spectral Deep Residual Convolutional Neural Network. IEEE Trans. Geosci. Remote Sens..

[28] Zhao W., Du S. (2016). Spectral–Spatial Feature Extraction for Hyperspectral Image Classification: A Dimension Reduction and Deep Learning Approach. IEEE Trans. Geosci. Remote Sens..

[29] Zhong Z., Li J., Luo Z., Chapman M. (2018). Spectral–Spatial Residual Network for Hyperspectral Image Classification: A 3-D Deep Learning Framework. IEEE Trans. Geosci. Remote Sens..

[30] Wang W., Dou S., Jiang Z., Sun L. (2018). A Fast Dense Spectral–Spatial Convolution Network Framework for Hyperspectral Images Classification. Remote Sens..

[31] Duan P., Kang X., Li S., Ghamisi P. (2019). Noise-Robust Hyperspectral Image Classification via Multi-Scale Total Variation. IEEE J. Sel. Top. Appl. Earth Obs. Remote Sens..

[32] Fang S., Quan D., Wang S., Zhang L., Zhou L. A Two-Branch Network with Semi-Supervised Learning for Hyperspectral Classification. Proceedings of the 38th IEEE International Geoscience and Remote Sensing Symposium (IGARSS).

[33] Zhang C., Li G., Du S. (2019). Multi-Scale Dense Networks for Hyperspectral Remote Sensing Image Classification. IEEE Trans. Geosci. Remote Sens..

[34] Yang J., Zhao Y., Chan J.C.-W., Yi C. Hyperspectral image classification using two-channel deep convolutional neural network. Proceedings of the 36th IEEE International Geoscience and Remote Sensing Symposium (IGARSS).

[35] He M., Li B., Chen H. Multi-scale 3D deep convolutional neural network for hyperspectral image classification. Proceedings of the IEEE International Conference on Image Processing (ICIP).

[36] Pooja K., Nidamanuri R.R., Mishra D. Multi-Scale Dilated Residual Convolutional Neural Network for Hyperspectral Image Classification. Proceedings of the 10th Workshop on Hyperspectral Imaging and Signal Processing: Evolution in Remote Sensing (WHISPERS).

[37] Wu S.F., Zhang J.P., Zhong C.X. Multiscale spectral-spatial unified networks for hyperspectral image classification. Proceedings of the IEEE International Geoscience and Remote Sensing Symposium (IGARSS).

[38] Fang B., Liu Y., Zhang H., He J. (2022). Hyperspectral Image Classification Based on 3D Asymmetric Inception Network with Data Fusion Transfer Learning. Remote Sens..

[39] Lu Z., Xu B., Sun L., Zhan T., Tang S. (2020). 3-D Channel and Spatial Attention Based Multiscale Spatial–Spectral Residual Network for Hyperspectral Image Classification. IEEE J. Sel. Top. Appl. Earth Obs. Remote Sens..

[40] Sun H., Zheng X., Lu X., Wu S. (2020). Spectral–Spatial Attention Network for Hyperspectral Image Classification. IEEE Trans. Geosci. Remote Sens..

[41] Hu J., Liu Y., Kang X., Fan S. (2022). Multilevel Progressive Network With Nonlocal Channel Attention for Hyperspectral Image Super-Resolution. IEEE Trans. Geosci. Remote Sens..

[42] Wang X., Girshick R., Gupta A., He K. Non-local Neural Networks. Proceedings of the 2018 IEEE/CVF Conference on Computer Vision and Pattern Recognition.

[43] Szegedy C., Liu W., Jia Y., Sermanet P., Reed S., Anguelov D., Erhan D., Vanhoucke V., Rabinovich A., Liu W. Going deeper with convolutions. Proceedings of the 2015 IEEE Conference on Computer Vision and Pattern Recognition (CVPR).

[44] Delibasoglu I., Cetin M. (2020). Improved U-Nets with inception blocks for building detection. J. Appl. Remote Sens..

[45] Zhang Z., Wu C., Coleman S., Kerr D. (2020). DENSE-INception U-net for medical image segmentation. Comput. Methods Programs Biomed..

[46] Halawa L.J., Wibowo A., Ernawan F. Face Recognition Using Faster R-CNN with Inception-V2 Architecture for CCTV Camera. Proceedings of the 3rd International Conference on Informatics and Computational Sciences (ICICoS).

[47] Chen J., Lin Y., Guo Y., Zhang M., Alawieh M.B., Pan D.Z. (2019). Lithography hotspot detection using a double inception module architecture. J. Micro/Nanolithogr. MEMS MOEMS.

[48] Purnamawati S., Rachmawati D., Lumanauw G., Rahmat R.F., Taqyuddin R. Korean letter handwritten recognition using deep convolutional neural network on android platform. Proceedings of the 2nd International Conference on Computing and Applied Informatics.

[49] Singh S., Kumar R. (2022). Breast cancer detection from histopathology images with deep inception and residual blocks. Multimed. Tools Appl..

[50] Shokri M., Harati A., Taba K. (2020). Salient object detection in video using deep non-local neural networks. J. Vis. Commun. Image Represent..

[51] Wang J., Qiao X., Liu C., Wang X., Liu Y., Yao L., Zhang H. (2021). Automated ECG classification using a non-local convolutional block attention module. Comput. Methods Programs Biomed..

[52] Wang S., Hou X., Zhao X. (2020). Automatic Building Extraction from High-Resolution Aerial Imagery via Fully Convolutional Encoder-Decoder Network with Non-Local Block. IEEE Access.

[53] Hyperspectral Remote Sensing Scenes[EB/OL]. http://www.ehu.eus/ccwintco/index.php?title=Hyperspectral-Remote-Sensing-Scenes.

[54] Hu J., Shen L., Sun G. Squeeze-and-Excitation Networks. Proceedings of the IEEE Conference on Computer Vision and Pattern Recognition (CVPR).

[55] Woo S., Park J., Lee J.Y., Kweon I.S. CBAM: Convolutional block attention module. Proceedings of the European Conference on Computer Vision.

[56] Ben Hamida A., Benoit A., Lambert P., Ben Amar C. (2018). 3-D Deep Learning Approach for Remote Sensing Image Classification. IEEE Trans. Geosci. Remote Sens..

[57] Paoletti M.E., Haut J.M., Fernandez-Beltran R., Plaza J., Plaza A.J., Pla F. (2019). Deep Pyramidal Residual Networks for Spectral–Spatial Hyperspectral Image Classification. IEEE Trans. Geosci. Remote Sens..

